# Multiparametric MRI and Computational Modelling in the Assessment of Human Articular Cartilage Properties: A Comprehensive Approach

**DOI:** 10.1155/2018/9460456

**Published:** 2018-05-15

**Authors:** J. Thüring, K. Linka, M. Itskov, M. Knobe, L. Hitpaß, C. Kuhl, D. Truhn, S. Nebelung

**Affiliations:** ^1^Department of Diagnostic and Interventional Radiology, Aachen University Hospital, Aachen, Germany; ^2^Department of Continuum Mechanics, RWTH Aachen University, Aachen, Germany; ^3^Department of Orthopaedic Trauma Surgery, Aachen University Hospital, Aachen, Germany

## Abstract

Quantitative magnetic resonance imaging (qMRI) is a promising approach to detect early cartilage degeneration. However, there is no consensus on which cartilage component contributes to the tissue's qMRI signal properties. *T*1, *T*1*ρ*, and *T*2^⁎^ maps of cartilage samples (*n* = 8) were generated on a clinical 3.0-T MRI system. All samples underwent histological assessment to ensure structural integrity. For cross-referencing, a discretized numerical model capturing distinct compositional and structural tissue properties, that is, fluid fraction (FF), proteoglycan (PG) and collagen (CO) content and collagen fiber orientation (CFO), was implemented. In a pixel-wise and region-specific manner (central versus peripheral region), qMRI parameter values and modelled tissue parameters were correlated and quantified in terms of Spearman's correlation coefficient *ρs*. Significant correlations were found between modelled compositional parameters and *T*1 and *T*2^⁎^, in particular in the central region (*T*1: *ρs* ≥ 0.7 [FF, CFO], *ρs* ≤ −0.8 [CO, PG]; *T*2^⁎^: *ρs* ≥ 0.67 [FF, CFO], *ρs* ≤ −0.71 [CO, PG]). For *T*1*ρ*, correlations were considerably weaker and fewer (0.16 ≤ *ρs* ≤ −0.15). QMRI parameters are characterized in their biophysical properties and their sensitivity and specificity profiles in a basic scientific context. Although none of these is specific towards any particular cartilage constituent, *T*1 and *T*2^⁎^ reflect actual tissue compositional features more closely than *T*1*ρ*.

## 1. Introduction

Osteoarthritis (OA) is a multifactorial and heterogeneous disease with a huge socioeconomic burden [1, 2]. Timely detection and treatment by chondroprotective agents, axis-modifying surgery, and lifestyle modifications provide the only widely acknowledged approach to reduce the disease burden in halting the degenerative cascade [3]. Although OA is a disease of the entire joint and involves intra- and extraarticular structures alike, cartilage degeneration is commonly defined as the central hallmark of the disease [4, 5]. More specifically, alterations in the extracellular cartilage matrix characterize early stages of cartilage degeneration [6] although macroscopically, the tissue appears unaltered [7].

Standard clinical diagnostic tools including morphological magnetic resonance imaging (MRI) fail to detect early cartilage degeneration [8, 9]. Quantitative MRI techniques (qMRI, syn. functional MRI) are commonly used to quantify and spatially map distinct tissue relaxivity characteristics. Despite encouraging preclinical and clinical results [9, 10], these techniques are also insufficiently sensitive and specific to detect early cartilage degeneration [11, 12]. Among the qMRI techniques available, *T*1*ρ*, *T*1, and *T*2^*∗*^ have been theorized to provide quantitative information on compositional and structural tissue properties beyond mere morphological imaging and are the subject of ongoing scientific research [13–15]. While some authors suggest that these parameters are indispensable in the early detection of cartilage degeneration [16, 17], others report low and diagnostically insufficient diagnostic performances [12, 18, 19].

Briefly, *T*1*ρ* is widely considered to provide a surrogate parameter of the tissue's macromolecular content. Based on the spin-lattice relaxation in the rotating frame, *T*1*ρ* relaxation is governed by the interaction of fluid-phase and solid-phase protons and has been demonstrated to be sensitive to water content and extracellular matrix properties [8, 20–24]. Yet, the exact structural or compositional correlate of *T*1*ρ* remains to be determined: Some studies suggested specificity for proteoglycans [20, 21], while others found various factors such as tissue hydration, collagen content, and orientation to be contributory, too [8, 22–24]. Nonetheless, common consensus prevails that *T*1*ρ* provides a marker of biologically meaningful intratissue changes [25–28].

Data on noncontrast enhanced *T*1 mapping in cartilage are sparse. Commonly applied in combination with gadolinium-enhanced imaging techniques such as dGEMRIC (delayed gadolinium-enhanced MRI of cartilage), nonenhanced *T*1 is considered to be primarily related to the PG content (as compared to the CO content) [29], while tissue hydration has also been reported to be relevant [30].* In vitro* [31] and* in vivo* studies [32] alike have reported *T*1 to be increased in structurally damaged cartilage, in particular in the presence of severe PG loss, cartilage edema, and surface disintegration.

*T*2^*∗*^ measures the loss of signal strength due to spin-spin interactions (i.e., *T*2 relaxation) and coherent dephasing effects secondary to magnetic field nonuniformity (i.e., susceptibility effects and scanner design imperfections). Although close technical connections exist between *T*2 and *T*2^*∗*^, fundamental and substantial differences become evident in variable degrees of cartilage degeneration [12, 17]. The characteristic signal decay thus detected is inherently different and presumably marked by different susceptibilities towards bulk water and macromolecular tissue properties. Nonetheless, the exact diagnostic value of *T*2^*∗*^ mapping in the comprehensive evaluation of cartilage remains to be defined [12, 33–36].

It is against this background that a true need exists to further define these parameters' sensitivity and specificity profiles in relation to distinct cartilage components such as fluid fraction (FF), collagen (CO), and proteoglycan (PG) content as well as collagen fiber orientation (CFO). One promising approach is to computationally model cartilage properties as a function of depth-dependent tissue properties. In an earlier study, our group investigated the pixel-wise correlation between *T*2 mapping (as a measure of tissue hydration and collagen content [8, 9, 11]) and computationally modelled tissue properties [37]. Based on this approach, the present study aimed to define the pixel-wise correlation between actual *T*1*ρ*, *T*1, and *T*2^*∗*^ maps and computationally modelled parameters in an effort to further define these parameters' specificity and sensitivity profiles. We hypothesized the qMRI and computationally modelled parameters to be significantly correlated with the modelled tissue parameters. To this end, a validated numerical model of human articular cartilage [37] and spatially resolved parameter maps of *T*1*ρ*, *T*1, and *T*2^*∗*^ relaxation properties [38] were therefore brought together to study the putative interrelatedness in a basic research context.

## 2. Materials and Methods

### 2.1. Study Design

This study was set up as a comparative study with an experimental and a computational modelling part. *T*1, *T*1*ρ*, and *T*2^*∗*^ maps of eight samples from a previously published cohort [38] were compared against a discretized computational modelling approach.

### 2.2. Experimental Part

Of a previously published cohort consisting of 20 osteochondral samples [38], eight samples were included in this study. To achieve geometrical and modelling consistency only histologically perfectly convex and structurally intact osteochondral samples from the lateral femoral condyles were included as before [37]. Correspondingly, flat, concave, or otherwise nonconvex samples were excluded. Please refer to [38] for a detailed outline of the superordinate in- and exclusion criteria.

In practical terms, the osteochondral samples were prepared in a standardized manner: After sterile excision during total joint replacement surgery, the samples were cut to standard size (length × width; ca. 1.5 × 1.5 cm) and the mid-sagittal planes (for the sake of reproducible plane definition) were defined by creating standardized notches at opposing sample sides. MRI measurements were performed on a clinical 3.0-T MRI system (Achieva, Philips, Best, Netherlands) using a modified single-channel prostate coil (BPX-30 endorectal coil, Medrad/Bayer, Leverkusen, Germany; stripped of its inflatable balloon) that was positioned to circumferentially comprise the customized sample container at the height of the cartilage layer for the optimized signal-to-noise ratio. Special care was taken to position the samples within the coil's isocenter and to align their mid-sagittal planes along and their surfaces parallel with the main magnetic field *B*_0_. Upon confirming the absence of substantial *B*_0_ inhomogeneity (by means of *B*_0_ mapping), proper sample placement was verified using axial and coronal scout views. In addition to a clinical standard *T*2 turbo spin echo sequence used for morphological evaluation (sagittal plane; Repetition time, 1000 ms; echo time, 35,4 ms; field of view, 42.0 × 27.3 mm; acquisition matrix, 160 × 162; reconstruction matrix, 224 × 224; flip angle, 90°; section thickness, 2 mm; number of signal averages, 2), *T*1, *T*1*ρ*, and *T*2^*∗*^ mapping of the mid-sagittal plane was performed using the sequence parameters given in Table 1. Quantitative parameter maps were generated by loading the MR raw data and each pixel's time constants into MATLAB (R2015a, TheMathWorks, Natick, MA, USA).

For each voxel, MRI parameters were quantified by fitting the measured signal intensities to the theoretical signal evolution in a least-squares manner on the basis of customized and standard fitting routines as provided in MATLAB. Fit quality was checked with *R*^2^ statistics adjusted to the degrees of freedom and optimization was considered appropriate if *R*^2^ ≥ 0.95. Theoretical signal evolutions are given in (1)–(3) below (i.e., *T*2^*∗*^ (1), *T*1*ρ* (2), and *T*1 (3)). Of note, *A* denotes the signal amplitude and *B* the noise floor levels. *T*_*E* denotes the echo time, *T*_SpinLock the duration of the spin lock pulse, TR the repetition time, and TI the inversion recovery time, that is, the time delay between the initial inversion recovery pulse and the read-out.

(1) SignalT_E=Aexp⁡−T_ET2∗

(2) SignalT_SpinLock=Aexp⁡−T_SpinLockT1ρ

(3) SignalT1=A1−2∗exp⁡−TIT1+exp⁡−TRT1.

Fit quality was ascertained using *R*-squared (*R*^2^) statistics adjusted to the degrees of freedom. Of note, only echo times < 60 ms were included in the *T*2^*∗*^ maps.

**Figure 1:**
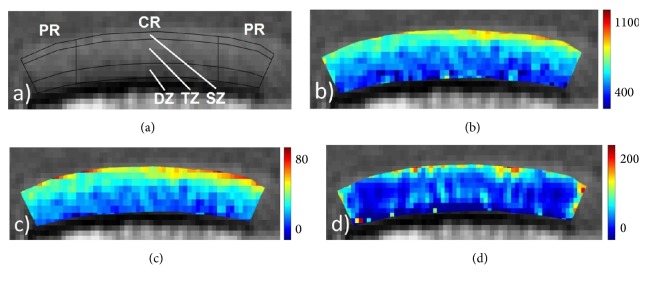
(a) *T*2-weighted morphological mid-sagittal image with a definition of the segmentation routine and the regions-of-interest: central region (CR, width of 6 mm); peripheral region (PR, adjacent to CR on both sample sides); superficial zone (SZ, 0–15% of sample depth); transitional zone (TZ, 15–65%); deep zone (DZ, 65–100%). (b–d) Corresponding *T*1 (b), *T*2^*∗*^ (c), and *T*1*ρ* (c) maps. Scales extend from 400 to 1100 ms (b), 0 to 80 ms (c), and 0 to 200 ms (d).

Sample-specific cartilage outlines were defined manually on the basis of the *T*2-weighted morphological images and validated against the *T*1, *T*1*ρ*, and *T*2^*∗*^ images. Regions-of-interest (ROIs) were defined regionally (peripheral region [PR], central region [CR]) and zonally (superficial zone [SZ], transitional zone [TZ], and deep zone [DZ]). Please see Figure 1 for more details.

**Figure 2:**
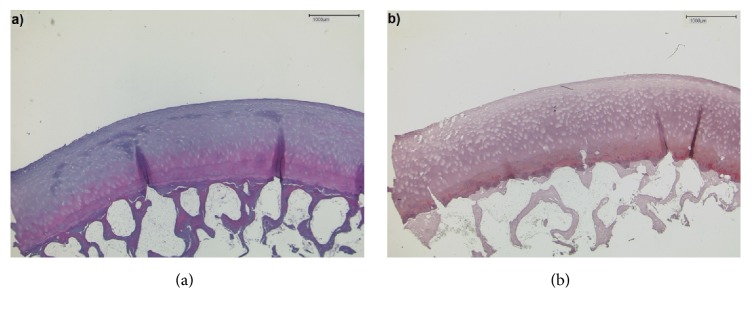
Corresponding histological sections of the sample shown in Figure 1. (a) Hematoxylin-eosin and (b) Safranin O staining. Slight discoloration upon proteoglycan staining and focal cell proliferation were noted. Mankin grade 0.

For reference purposes, samples underwent histological analysis according to standard routines, that is, decalcification/fixation, embedding in paraffin, cutting to histological sections, staining with hematoxylin-eosin, and Safranin O and imaging/documentation using a light microscope (DM/LM-P, Leica, Wetzlar, Germany). Histological grading was performed according to the Mankin classification, which is a semiquantitative histological grading scheme used to assess cartilage degeneration [39]. Structural, cellular, proteoglycan staining-associated, and tidemark integrity-associated tissue properties were scored separately and summed up to give the Mankin sum score (range, 0–14), which was used to grade the sample as normal (Mankin sum scores 0–4, Mankin grade 0), mildly (Mankin sum scores 5–8, Mankin grade I), moderately (Mankin sum scores 9-10, Mankin grade II), and severely degenerated (Mankin sum scores 11–14, Mankin grade III) (Figure 2).

### 2.3. Computational Modelling

Cartilage tissue parameters were modelled using a sample-specific discretization of the mid-sagittal imaging plane of each sample to rectangular elements. In this regard, each element was associated with the local cartilage properties in a depth-dependent manner. Please refer to Linka et al. [37] for more details.

In brief, the cartilage tissue was considered a fluid-filled solid matrix consisting of collagen (CO) fibers and negatively charged proteoglycan (PG) aggregates [40, 41]. Thus, the volume fractions satisfied the condition

(4) $$\begin{array}{c} \overset{3}{\underset{\eta }{\sum }}\phi_{\eta }=1,\;\eta \in \left(\text{fluid},CO,PG\right), \end{array}$$

where *ϕ*_fluid_, *ϕ*_CO_, and *ϕ*_PG_ denoted the fluid, CO, and PG volume fractions. The solid volume fraction *ϕ*_*s*_ was inferred as

(5) $$\begin{array}{c} \phi_{s}=1-\phi_{\text{fluid}}. \end{array}$$

**Figure 3:**
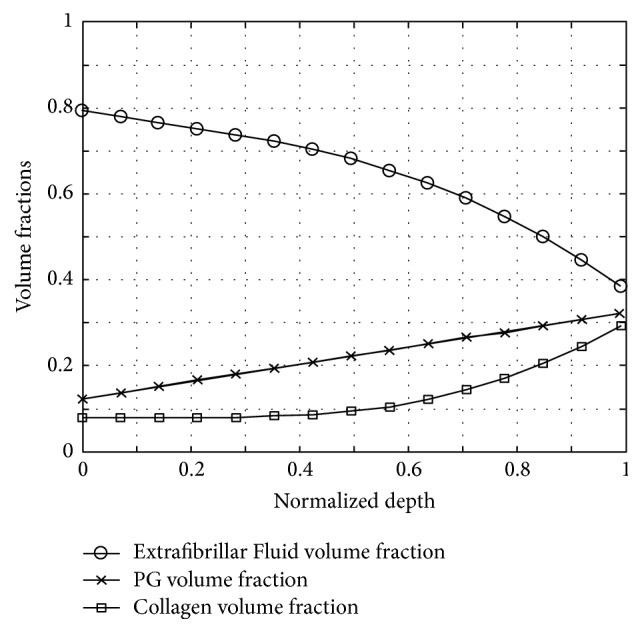
Cartilage volume fractions of extrafibrillar fluid (circles), proteoglycans (crosses), and collagen (boxes) as functions of the normalized sample depth. The depth-dependent tissue properties were adapted from Wilson et al. (see main text for more details).

The required information on the tissue's composition was adapted from [40–42] and may be plotted as volume fractions as functions of the normalized sample depth (Figure 3).

**Figure 4:**
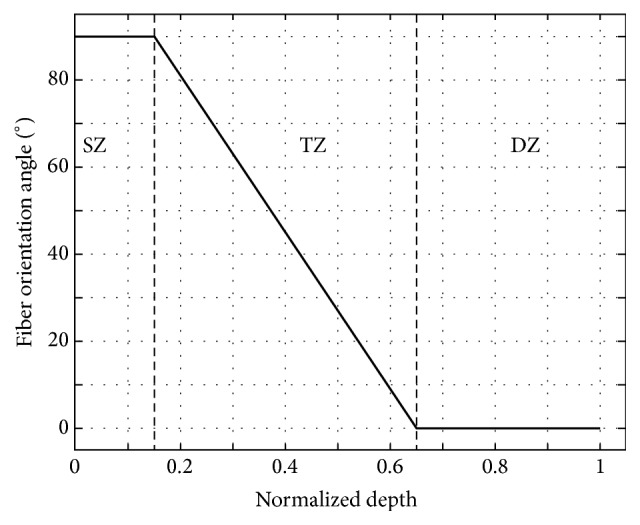
Mean fiber orientation angle [°] as a function of the normalized cartilage depth. The entire cartilage depth was structured into three zones: superficial zone (SZ; 0–15%), transitional zone (TZ, 15–65%), and deep zone (DZ, 65–100%).

In line with the idealized arcade model proposed by Benninghoff [43], the local collagen fiber orientation (CFO) angle *ϑ*_fib_(*z*) was plotted relative to the subchondral layer's orientation. As a function of the normalized cartilage depth, *ϑ*_fib_(*z*) was implemented in a depth-dependent manner, that is, set to 0° in the deep zone and to 90° in the superficial zone and approximated by a linear function in-between (Figure 4). Of note, the surface configuration was determined based on the curvature of each individual sample as indicated by the MR images.

In addition and in line with the Wilson model, the rotational symmetry of each fiber family (as defined by Benninghoff [43]) was taken into account by implementing eight local directions of fiber family orientation (*n* = 8) equiangular to the surface [42].

### 2.4. Statistical Analysis

Statistical analyses were performed using GraphPad Prism (version 5.0; GraphPad, San Diego, CA, USA). QMRI parameters and modelled tissue composition parameters were comparatively evaluated in a pixel-wise manner and calculated for the peripheral and central regions, respectively, and subsequently quantified using Spearman's correlation coefficient *ρ*_s_. Best-fit analyses were performed by minimizing *R*-squared (*R*^2^): to this end, volume fractions were fitted against the exponential function *a*_*x*_exp⁡(*b*_*x*_*ϕ*_*x*_) + *c*_*x*_, where *x* = PG, CO, FF and *ϕ*_*x*_ denotes the respective volume fraction, while *a*_*x*_, *b*_*x*_, *c*_*x*_ represent the associated parameters. In contrast, the CFO was fitted against the sinusoidal function *a*_CFO_sin⁡(*b*_CFO_*θ*_CFO_) + *c*_CFO_, where *θ*_CFO_ denotes the fiber orientation. Note that the utilized fitting functions were chosen by evaluating the best results in terms of *R*^2^.

Moreover, the qMRI parameters were determined for the individual regions and zones, output as mean ± standard deviation (*M*  ± SD) and assessed for significant differences using the Mann–Whitney *U* test and Kruskal-Wallis test, respectively, followed by Dunn's post hoc test wherever appropriate. The level of significance was set to *p* < 0.05. Data are presented as *M*  ± SD or *ρ*_*s*_ (*p* value).

## 3. Results

Mean regional and zonal pixel numbers for the qMRI maps were 63 ± 17 (SZ-CR), 214 ± 58 (TZ-CR), 126 ± 30 (DZ-CR), 36 ± 22 (SZ-PR), 106 ± 65 (TZ-PR), and 50 ± 32 (DZ-PR).

Mean regional and zonal qMRI parameter values are given in Table 1.

Of note, no significant differences were found upon regional assessment of the distinct tissue zones [data not shown]. In contrast, significant differences upon zonal assessment were found for *T*1 only. Post hoc analysis revealed these differences to be significant between the DZ and the other zones: DZ versus SZ and DZ versus TZ (Table 2).

The mean computationally modelled cartilage tissue parameters as a function of different regions and zones are given in Table 3.

Zonal and regional correlation analyses revealed strong and highly significant correlations between the modelled compositional parameters and *T*1 as well as *T*2^*∗*^, while considerably less numerous and weaker correlations were determined for *T*1*ρ* (Table 4).

**Figure 5:**
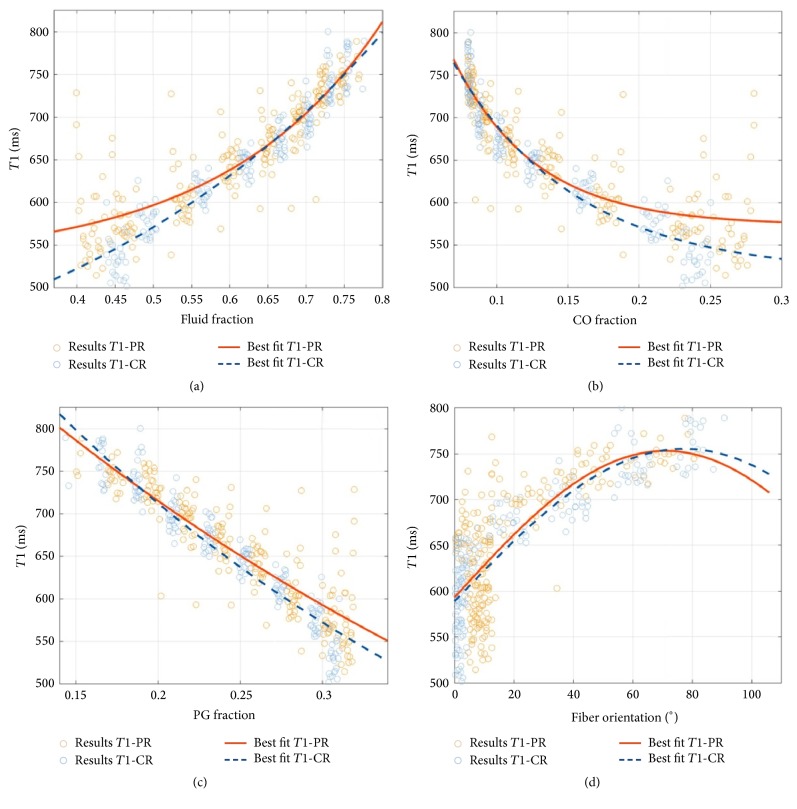
Correlation plots of mean *T*1 relaxation times and the FF (a), CO (b), and PG (c) volume fractions and the fiber orientation (d) on a per-pixel basis. Regional evaluation was performed for the peripheral region (PR, orange) and the central region (CR, blue). Read plots as follows: An individual data point indicates a single pixel's modelled quantity of the tissue property and the respective *T*1 relaxation time. Of note, the fiber orientation angles are given with respect to the cartilage normal.

For *T*1, moderate-to-strong and highly significant correlations were found between all four modelled tissue parameters. In terms of Spearman's correlation coefficient *ρ*_*s*_, correlations were stronger in the CR than in the PR. Regardless of the tissue region, correlations were distinctly positive for *T*1 versus FF and *T*1 versus CFO, while they were distinctly negative for *T*1 versus PG and *T*1 versus CO.  Figure 5 allows a more detailed appreciation of the statistical relationships. Best-fit analyses revealed these correlations to be exponential with higher *T*1 values indicating higher fluid fractions and collagen fiber angles (i.e., more perpendicular angles with regard to the cartilage surface). Correspondingly, higher *T*1 values were indicative of lower PG and CO contents with the former association appearing more exponential and the latter more linear.

**Figure 6:**
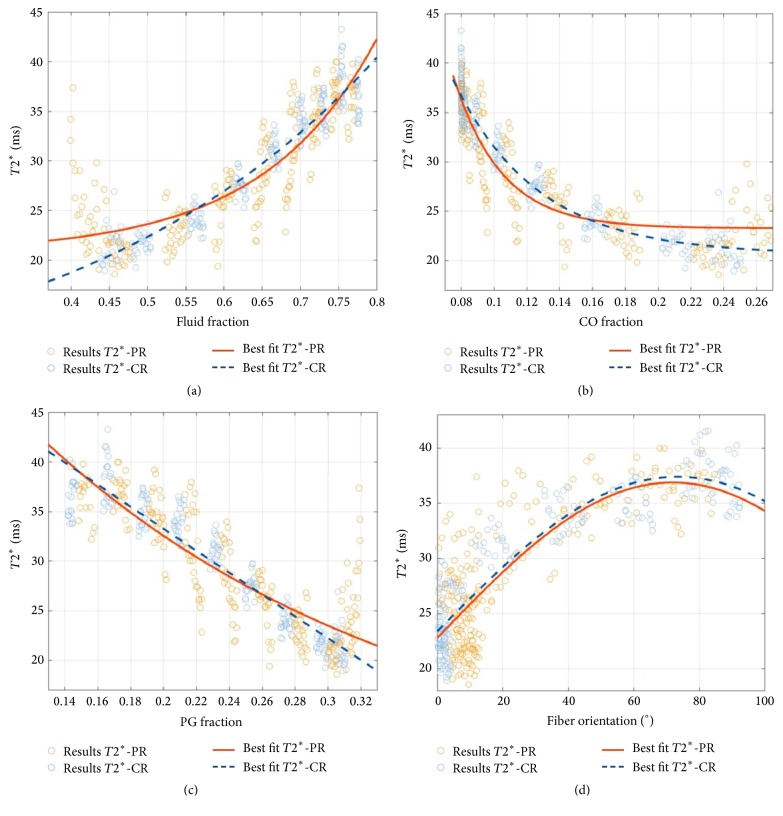
Correlation plots of mean *T*2^*∗*^ relaxation times and the FF (a), CO (b), and PG (c) volume fractions and the fiber orientation (d) on a per-pixel basis. Please refer to Figure 5 for a more detailed explanation of the graphs.

Overall, similar observations as for *T*1 were made for *T*2^*∗*^; that is, moderate-to-strong and highly significant correlations were found between all four modelled tissue parameters and *T*2^*∗*^. Likewise, correlations were stronger in the CR than in the PR.  Figure 6 gives a detailed representation of the statistical relationships.

**Figure 7:**
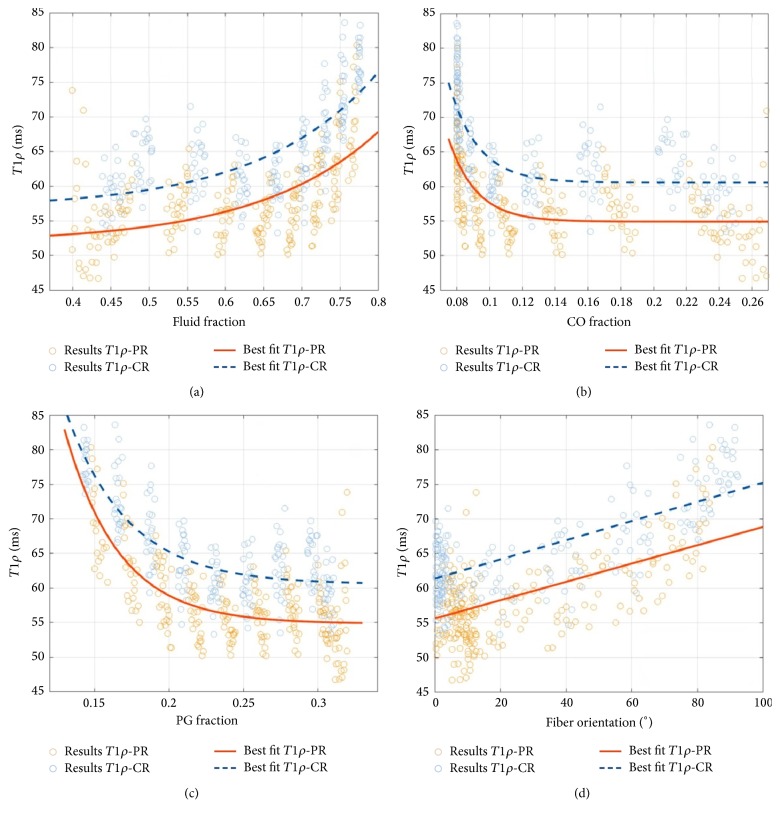
Correlation plots of mean *T*1*ρ* relaxation times and the FF (a), CO (b), and PG (c) volume fractions and the fiber orientation (d) on a per-pixel basis. Please refer to Figure 5 for a more detailed explanation of the graphs.

In contrast to *T*1 and *T*2^*∗*^, only weak correlations were found between *T*1*ρ* and the modelled tissue parameters. With the exception of CFO (that was weakly yet significantly and positively correlated in the CR and PR alike), correlations were significant in the CR only.  Figure 7 gives a detailed representation of the statistical relationships that were primarily found to be exponential except for the more linear association of *T*1*ρ* versus CFO.

## 4. Discussion

This study's most important finding is that computationally modelled parameters of cartilage composition, that is, FF, CO, PG, and CFO, are strongly correlated with *T*1 and *T*2^*∗*^, while they are only weakly correlated with *T*1*ρ*. These observations help to further refine each qMRI parameter's relevance in the assessment of cartilage and its pathologies, in both a scientific and a clinical context.

*T*1 and *T*2^*∗*^ were significantly and moderate-to-strongly correlated with the modelled tissue features, that is, positively correlated with the FF and the CFO and inversely correlated with the CO and PG contents. Even though the exact determinant of *T*1 relaxivity remains unclear and widely disputed [29], some authors consider *T*1 to be a marker of the tissue's hydration rather than any particular solid component, that is, PG and CO [30]. In contrast, other studies found the solid cartilage components to be more relevant to *T*1 relaxation characteristics, in particular in the assessment of tissue functionality [44, 45]. *T*1 relaxation in cartilage is largely unaffected by the tissue's orientation in the magnet [46]. On a molecular scale, *T*1 relaxation indicates the very slow motion of water molecules secondary to the constraining effects of the proteoglycans within the solid matrix and the retention forces thus created [47]. In terms of the respective correlation coefficients, our study suggests that all three cartilage constituents contribute to *T*1 relaxation, regardless of the tissue region examined. However, as *T*1 values declined significantly towards the deep cartilage zone, which is well in line with the literature [47, 48], each constituent's exact contribution to *T*1 relaxation characteristics is likely to be variable with differing cartilage depths. As indicated by the Wilson model [40, 41], the superficial zone is characterized by the highest water and the lowest PG and CO contents, while this ratio changes considerably towards deeper tissue zones so that the cartilage-bone transition is characterized by a nearly evenly balanced ratio of the three constituents. Thus, our correlation findings of higher *T*1 values (being reflective of higher FF as well as lower CO and PG contents) in the SZ and lower *T*1 values in the TZ and DZ fit well and render our results plausible. Moreover, significant positive correlations were found for *T*1 and CFO. Superficial zone collagen fibers are oriented parallel to the surface, while they change course in the transitional zone to be perpendicular to the surface in the deep zone [43]. As *T*1 is known to be isotropic to the magnetic field direction, that is, uniform in all orientations, one should be cautious in drawing biologically meaningful conclusions as the observed statistical relationship may first and foremost reflect the other modelled constituents' depth-dependent properties. In summary, even though significant correlations between *T*1 and compositional cartilage properties have been found, *T*1 alone seems to be too unspecific and should therefore not to be used as a stand-alone parameter in the evaluation of cartilage tissue properties.

Overall, similar findings as for *T*1 were made for *T*2^*∗*^. Similarly, *T*2^*∗*^ values tended to be higher in the SZ and TZ than in the DZ, which is well in line with literature findings by our group and others [12, 14, 33, 38]. Technically, *T*2^*∗*^ relaxation refers to the immediate signal loss following the initial excitation pulse that, along with the spin-spin interactions governing *T*2 relaxation, is due to magnetic field inhomogeneities within each voxel. Characteristic of all gradient echo sequences, *T*2^*∗*^ mapping makes use of magnetic susceptibility effects of inherently inhomogeneous tissues. In cartilage, *T*2^*∗*^ mapping is considered to be reflective of collagen microstructure and its changes rather than collagen content [17, 34, 49, 50]. However, our data indicate that no such specificity may be assumed as all modelled compositional tissue features displayed highly significant correlations with *T*2^*∗*^. Moreover, *T*2^*∗*^ and *T*2 are sensitive markers of water content and interactions between water and collagen [17] and indirectly reflect collagen fiber orientation within cartilage [33, 34], while, to our best knowledge, no data are available on the technique's specificity profile in relation to the solid matrix components. Our data indicate an inverse statistical relationship, that is, low *T*2^*∗*^ values being indicative of high CO and PG contents (and vice versa), even though each cartilage constituent's exact contribution to the *T*2^*∗*^ relaxation characteristics remains hypothetical at the moment. These findings warrant further basic research, in particular as the exact determinant and the true diagnostic value of *T*2^*∗*^ in the graduation of cartilage degeneration remain the topic of ongoing scientific discussions [12, 14, 33, 36].

For *T*1*ρ*, only weak correlations were found that were primarily significant in the central region. *T*1*ρ* relaxation (commonly referred to as the* spin-lattice relaxation in the rotating frame*) is determined by applying spin lock pulses of variable durations (i.e., low-powered radiofrequency pulses) to the magnetization in the transverse plane, thereby locking the magnetization in the transverse plane so that it interacts with the surrounding environment [51]. Hence, *T*1*ρ* is sensitive for the low-frequency motional interactions between the tissue's macromolecules and bulk water, that is, the local macromolecular environment of the cartilage extracellular matrix, while it is considerably less sensitive to the local fibril orientation [47]. Some early studies reported specificity for PGs [20, 21], while nowadays, common consensus prevails that several factors are contributing to *T*1*ρ* relaxation characteristics [8, 22–24]. Using suspensions of variable concentrations of PGs and CO to determine the effect of concentration on *T*1*ρ* relaxation, Menezes et al. found similar exponential decreases as in our study [23]. These findings were confirmed* in vivo* by Nishioka et al. and Wong et al. who found significant inverse correlations between *T*1*ρ* and PG content (*r* = −0.64, *p* < 0.001 [52]; *r* = −0.20, *p* = 0.025 [53]). Thus, higher *T*1*ρ* values indicate lower concentrations of the solid components and vice versa, which was confirmed by inverse (yet weak) correlations in our study. Weakly positive correlations were found for FF and CFO (though significant in the central region only), which confirms earlier findings by Nishioka et al. [52] and Xia [47] who reported limited correlation between *T*1*ρ* and water content as well as low sensitivities of *T*1*ρ* to the local fibril orientation. In summary, *T*1*ρ* seems to be as unspecific as *T*1 or *T*2^*∗*^, yet its correlation profile suggests that this parameter is not suitable to quantify any particular cartilage constituent but rather provides a surrogate parameter for the individual tissue constituents' main interactions including scalar coupling, dipole-dipole interactions, and chemical exchange processes [9, 54–56]. It remains to be seen to what extent *T*1*ρ* really helps to characterize the tissue's macromolecular configuration and their changes in health and disease.

For all qMRI parameters, stronger correlations were found in the central than in the peripheral tissue regions, which is primarily due to the sample's specific geometry. Only perfectly convex and structurally intact osteochondral samples from the lateral femoral condyles were included. With a mean curvature of the femoral condyle of 4.4/m [57], considerable variability in the angular orientation of the collagen fiber bulk in relation to the main magnetic field *B*_0_ needs to be assumed and is most likely responsible for the observed regional differences. This of course is due to the fact that all qMRI parameters are anisotropic (except for *T*1) and display different depth-dependent parameter profiles at different sample orientations relative to *B*_0_ [47].

Our study is characterized by a number of limitations. For once, only eight perfectly convex samples (of the original 20 samples) were included in this study for reasons of geometrical and modelling consistency, which limits its overall power. Thus, future studies using similar modelling approaches should be conducted on less uniform and more polymorphous sample configurations to assess the reproducibility of our findings, which is a prerequisite to be able to study more complex and anatomical cartilage configurations within the joint. Another aspect to consider is the* in vitro*-character of our study with the potential introduction of yet ill-controlled variables such as tissue harvest and prolonged storage outside the joint, which limits the overall* in vivo *transferability of our findings.

Another limitation relates to the input parameters utilized for the computational model. The tissue compositional properties and the depth-wise fiber architecture were derived from general assumptions of the tissue's overall configuration [40, 41, 43]. Although our results indicate that qMRI parameters can be used to assess compositional and structural cartilage properties, the complexities of all possible interactions and interdependencies between the qMRI parameters and their exact cartilage correlates (as assessed by our idealized computational model) remain to be defined in future studies. Future studies should therefore include extensions of the underlying model, for example, by sample-specific and spatially resolved measures of tissue structure and composition for the sake of more accurate correlation purposes. In this regard, Fourier Transform Infrared Spectroscopy (FTIR), Polarized Light Microscopy (PLM), or Diffusion-Tensor MRI (DT-MRI) could provide potentially interesting methods for comparative assessment.

## 5. Conclusion

To our best knowledge, this study is the first of its kind that brings together a sample-specific computational model of human articular cartilage and multiparametric quantitative MRI in a histologically controlled experimental setting. In a basic scientific context, each qMRI parameter is further characterized in its biophysical properties as well as in its sensitivity and specificity profile towards cartilage structural and compositional properties. *T*1 and *T*2^*∗*^ reflect actual tissue compositional features more closely than *T*1*ρ*, even though no parameter is specific towards any particular cartilage constituent.

## Figures and Tables

**Table 1:** Detailed acquisition parameters of MR sequences.

Sequence (Type)	Sequence parameters
*T*1 (inversion recovery)	Repetition time, 3000 ms; turbo spin-echo factor, 5; inversion time, 150, 300, 500, 800, 1000, 1300, and 1500 ms; field of view, 42.0 × 27.3 mm; acquisition matrix, 160 × 154; reconstruction matrix, 224 × 224; flip angle, 90°; section thickness, 2 mm; number of signal averages, 1

*T*1*ρ* (spin-lock multigradient echo)	Repetition time/echo time, 30 ms/4.2 ms; spin-lock durations, 0/10/20/30/40 ms; readout parameters: field of view, 42.0 × 27.3 mm; acquisition matrix, 160 × 98; reconstruction matrix, 224 × 224; flip angle, 11°; section thickness, 2 mm; number of signal averages, 4

*T*2^*∗*^ (multigradient echo)	Repetition time, 300 ms; echo time, 3.9 ms + *n* × 6.9 (*n* = 0–14); field of view, 42.0 × 27.3 mm; acquisition matrix, 160 × 162; reconstruction matrix, 224 × 224; flip angle, 38°; section thickness, 2 mm; number of signal averages, 6

**Table 2:** Mean regional and zonal qMRI parameter values [ms]. Mean ± standard deviation. Regional and zonal assessment was performed using the Mann–Whitney *U* test and Kruskal Wallis test, respectively. While no significant differences were found upon regional assessment [data not shown], significant differences were found upon zonal assessment and are printed in *bold-type*. See Figure 1 for a definition of the different regions (PR, CR) and zones (SZ, TZ, DZ).

	PR			CR		
	*T*1 [ms]	*T*1*ρ* [ms]	*T*2^*∗*^ [ms]	*T*1 [ms]	*T*1*ρ* [ms]	*T*2^*∗*^ [ms]
SZ	682 ± 77	63 ± 53	29 ± 63	675 ± 94	79 ± 62	35 ± 53
TZ	700 ± 29	52 ± 21	29 ± 17	701 ± 41	61 ± 26	32 ± 20
DZ	585 ± 6	51 ± 9	22 ± 5	589 ± 13	58 ± 10	24 ± 7
*p* value	**0.017**	0.512	0.110	**0.042**	0.652	0.054
Post hoc details	TZ vs DZ			TZ vs DZ		
	SZ vs DZ			SZ vs DZ		

**Table 3:** Mean modelled tissue compositional parameters. Mean ± standard deviation. Of note, the modelling approach was equal for the central and the peripheral regions. Volume fraction of fluid (FF), collagen (CO) and proteoglycan contents (PG), and mean collagen fiber orientation angle (CFO). See Figure 1 for an explanation of the remaining abbreviations.

	FF [%]	CO [%]	PG [%]	CFO [°]
SZ	78 ± 11	8 ± 1	14 ± 6	90 ± 0
TZ	70 ± 9	9 ± 1	20 ± 9	45 ± 32
DZ	51 ± 7	20 ± 3	29 ± 11	0 ± 0

**Table 4:** Spearman's correlations between the modelled compositional and qMRI parameters in the separate regions (i.e. PR and CR) as quantified by Spearman's correlation coefficient *ρ*_*s*_. Significant correlations are printed in *bold-type*, while the levels of significance are further stratified as 0.01 < *p* ≤ 0.05 ( ^*∗*^), 0.001 < *p* ≤ 0.01 ( ^*∗∗*^), and *p* ≤ 0.001 ( ^*∗∗∗*^) and written in parentheses.

	PR			CR		
	*T*1	*T*1*ρ*	*T*2^*∗*^	*T*1	*T*1*ρ*	*T*2^*∗*^
FF	**0.66 (** ^**∗∗∗**^**)**	0.16	**0.55 (** ^**∗**^**)**	**0.8 (** ^**∗∗∗**^**)**	**0.15 (** ^**∗**^**)**	**0.71 (** ^**∗∗∗**^**)**
CO	**−0.65 (** ^**∗∗∗**^**)**	−0.15	**−0.55 (** ^**∗**^**)**	**−0.81 (** ^**∗∗∗**^**)**	**−0.13 (** ^**∗**^**)**	**−0.73 (** ^**∗∗∗**^**)**
PG	**−0.66 (** ^**∗∗∗**^**)**	−0.16	**−0.55 (** ^**∗**^**)**	**−0.8 (** ^**∗∗∗**^**)**	**−0.15 (** ^**∗∗**^**)**	**−0.71 (** ^**∗∗∗**^**)**
CFO	**0.5 (** ^**∗∗∗**^**)**	**0.16 (** ^**∗**^**)**	**0.44 (** ^**∗∗**^**)**	**0.7 (** ^**∗∗∗**^**)**	**0.16 (** ^**∗∗**^**)**	**0.67 (** ^**∗∗∗**^**)**
